# Analysis of resilience and sexual behavior in persons with HIV infection

**DOI:** 10.1186/s41155-017-0076-6

**Published:** 2017-10-03

**Authors:** Ludgleydson Fernandes de Araújo, Inmaculada Teva, José Hernández Quero, Antonio Ortega Reyes, María de la Paz Bermúdez

**Affiliations:** 1 Department of Psychology, Universidade Federal do Piauí, Campus Ministro Reis Velloso, Av. São Sebastião, 2819, Parnaíba, PI 64202-020 Brazil; 2 Mind Brain and Behavior (Spanish acronym CIMCYC) Research Center, University of Granada, Granada, Spain; 3 Faculty of Medicine, San Cecilio University Hospital, University of Granada, Granada, Spain

**Keywords:** Socio-demographic, Resilience, Sexual behavior, Persons with HIV infection

## Abstract

The main objective of this study was to evaluate ex post facto resilience in persons with HIV infection and its relationship to socio-demographic and sexual behavior variables. Participants included 159 persons with HIV infection, of both sexes, aged between 19 and 55 years. Fifty-one percent of patients were infected through homosexual means. Sixty-seven percent were in the asymptomatic phase of infection. Assessment instruments used were the following: a questionnaire on socio-demographic data and sexual behavior and the Connor-Davidson Resilience Scale. The evaluation was individual, voluntary, and anonymous. The results showed that 49.05% of patients had average resilience, 27.68% had high resilience, and 23.37% had low resilience. They found that heterosexual patients infected with HIV, diagnosed between 1985 and 1990 (23 and 28 years of diagnosis) and those who had disclosed their HIV status to more than 30 people, had greater resilience than homosexual patients, diagnosed between 1996 and 2000 (13 and 17 years of diagnosis) and those who had disclosed their HIV status to 1–5 people. Finally, resilience was not a predictor of sexual risk factor. It is suggested that health interventions take into account the resilience and psychological variables that may be beneficial to improve coping with the disease.

## Background

Over the last two decades, researchers have shown greater interest in studying resilience (Carvalho, Morais, Koller, & Piccinini, 2007). This psychological construct has been defined as the patterns of positive adaptation to risk and adversity (Liebenberg & Ungar, 2009; Masten & Cicchetti, 2012; Rutter, 2006), being understood as a process mediated by individual, family, and social factors for overcoming challenging situations (Cameron, Ungar, & Liebenberg, 2007; Masten & Tellegen, 2012) and the capacity to react positively to adverse and traumatic events (Orton, Griffiths, Green, & Waterman, 2012), in such a way that the individual gains additional protection and coping skills (Poletto & Koller, 2006). For building resilience, it is therefore necessary for the person to be exposed to difficult situations that jeopardize his/her physical and/or mental health (De Santis, Florom-Smith, Vermeesch, Barroso, & DeLeon, 2013). Taking resilience to be a process of interaction between the individual and the context in which he/her finds him/herself, two stages can be discerned. In the first stage, considered the acute stage, the problem situation causes the person to perceive a threat and learn to control this situation. Once the person has this new situation under control, the second stage takes place, in which the person must reorganize his/her life by including in it the changes created by the situation. The outcome of this is resilience (Fine, 1991).

In this study, resilience is defined as the individual’s ability to overcome adversity or psychosocial risks (Rutter, 1999). According to this author, resilience is not solely based on positive traits of the individual; a series of psychological determinant factors should be analyzed. Psychology believes that resilience is linked to personal resources, which lead to adaptive behaviors such as self-esteem, self-efficacy, and problem-solving ability, which act as protectors against adversity (Rutter, 1987).

The social and academic relevance of this study is primarily due to the lack of studies on the relationship between resilience and HIV infection (Araújo, Teva, & Bermúdez, 2015) and ways of coping with this disease. Secondly, this study has added ways of detecting levels of resilience and its relationship with psychosocial variables.

The majority of studies on resilience have focused on persons with physical and psychological problems and/or disorders such as stress (Braun-Lewensohn & Sagy, 2013), spouses who died of AIDS (Yu, Chan, Zhang, & Stewart, 2016), transgender women (Perez-Brumer et al., 2017), anxiety (Nabors et al., 2013), coping and resilience (Tippens, 2017), trauma (Pooley, Cohen, O’Connor, & Taylor, 2013), resilience and mastery among HIV-positive older gay and bisexual men (Emlet, Shiu, Kim, & Fredriksen-Goldsen, 2017), quality of life of gay, bisexual men and people/persons living with HIV (PLHIV) (Remor & Ulla, 2002), and disclosure of HIV status and psychological well-being (Zea, Reisen, Poppen, Bianchi, & Echeverry, 2005). A longitudinal study showed that internalized stigma and HIV diagnosis were associated with ways of coping (resilience) among PLHIV (Garrido-Hernansaiz, Murphy, & Alonso-Tapia, 2017). However, not many studies have been carried out on PLHIV.

Living with HIV infection and, prior to that, its diagnosis give rise to circumstances with significant physical and psychological repercussions that the infected person has to cope with (Carvalho et al., 2007). HIV infection, today considered as a chronic disease thanks to the development of and advances in antiretroviral therapies (Deeks, Lewin, & Havlir, 2013), presents a series of characteristics which make it different from other diseases and particularly adverse for the seropositive person. For one thing, most cases of HIV infection are caused by sexual risk behaviors, and for another, even today, seropositive individuals are still the object of stigma, discrimination, and marginalization by society (De Santis et al., 2013). Studies on resilience in the HIV/AIDS context have concluded that the higher the resilience, the higher the perception of quality of life and the lower the mental distress (see for example Faber, Shwartz, Schaper, Moonen, & McDaniel, 2000). Similarly, other studies have shown that resilience is positively associated with better cognitive coping, wellbeing, and acceptance of being HIV-positive (Munro & Edward, 2008; Orton et al., 2012). The study of resilience among these population is, therefore, particularly relevant but, in turn, lacking (De Santis et al., 2013).

As regards the link between resilience and socio-demographic variables, it has been shown that HIV-positive women with low education levels, low income, and no employment had high levels of resilience (Dale et al., 2014). Other studies have concluded that resilience was lower among asymptomatic seropositive males with lower levels of education and limited economic means (Lima, Azevedo, Amorim, & Saldanha, 2014).

Among the sexual behaviors and risk factors for becoming infected with HIV and other sexually transmitted infections (STI) are sexual intercourse without using a condom, starting sexual relations at a young age, and having multiple sexual partners (Teva, Bermúdez, Ramiro, & Ramiro-Sanchez, 2013). Along these lines, some researchers have emphasized the study of resilience as a new direction in which to orient efforts to prevent HIV infection (Yuen et al., 2013) and, similarly, other STI. For their part, Kurtz, Buttram, Surratt, and Stall (2012) concluded that resilience could be a key variable in actions aiming to reduce sexual risk behaviors among men who have sex with other men. So, we can hope that resilience might act as a factor protecting against the propagation of sexual risk behaviors. Bearing in mind that there are no studies which analyze the relationship between resilience and sexual risk behaviors in persons with HIV infection, it would be a valuable study area for contributing information of use for the prevention of STI/HIV: on the one hand, for preventing patients from contracting an STI in the first place and, on the other, to prevent them from passing it on to other people. In addition, analyzing resilience among PLHIV would enable actions to be designed for improving the psychological health and strategies for coping with this disease among these population.

Based on what we have set out above, we propose this study. Its aims are as follows: (1) to assess resilience and its relationship with socio-demographic variables and (2) to study whether resilience is a predictor of sexual risk behaviors among PLHIV.

## Methods

### Participants

The sample comprises 159 PLHIV, aged between 19 and 55 years old (*M* = 40.09 years old; SD = 9.05). Fifty-one percent of cases of infection were contracted homosexually, 43% heterosexually, and 6% due to sharing needles for injecting narcotics. The sample size was determined by the availability of PLHIV to voluntarily and anonymously participate in the study. The sample was classified according to the Centers for Disease Control and Prevention classification system for HIV infection (CDC, 1993); 67% were in the asymptomatic stage of infection (A—asymptomatic, acute HIV, or persistent generalized lymphadenopathy), 10% were in the symptomatic stage (B—symptomatic conditions, not A or C), and 23% were in the AIDS stage (C—AIDS-indicator conditions). Other socio-demographic features of the sample are shown in Table 1 **.**

**Table 1:** Socio-demographic characteristics of participants

Features	PLHIV (*N* = 159)
	*n* (%)
Sex	
Male	122 (77.00)
Female	37 (23.00)
Marital status	
Single	79 (50.00)
Married	31 (19.00)
Common-law spouse	22 (14.00)
Separated or divorced	14 (9.00)
Widow	13 (8.00)
Level of education	
No schooling	6 (4.00)
Primary education	50 (31.00)
Secondary education	62 (39.00)
University education	41 (26.00)
Sexual orientation	
Heterosexual	66 (41.00)
Homosexual	93 (59.00)
Age	
19 to 29 years old	23 (15.00)
30 to 42 years old	66 (41.00)
43 to 55 years old	70 (44.00)
Income	
Less than 600 euros	30 (19.00)
600 to 900 euros	27 (17.00)
900 to 1200 euros	30 (19.00)
More than 1200 euros	72 (45.00)
Current employment situation	
Employed	90 (57.00)
Unemployed	45 (28.00)
On sick leave	24 (15.00)
Year of HIV/AIDS diagnosis	
1985–1990	21 (13.00)
1991–1995	29 (18.00)
1996–2000	18 (11.00)
2001–2005	29 (18.00)
2006–2010	62 (40.00)
Persons to whom HIV status disclosed	
None	14 (9.00)
1 to 5 persons	78 (50.00)
6 to 15 persons	39 (24.00)
16 to 30 persons	12 (7.00)
More than 30 persons	30 (10.00)

### Design

This is a descriptive study using cross-sectional surveys.

### Instruments

#### Questionnaire on socio-demographic data and sexual behavior

Questions were devised to collect socio-demographic information (age, sex, marital status, sexual orientation, education level, household income, current employment situation, year in which diagnosed with HIV/AIDS, number of persons to whom HIV/AIDS status disclosed). To devise the questions on sexual behavior, previous research was taken into account (Teva et al., 2013). The participants’ stage of HIV infection was obtained from medical and clinical records at the Hospital of the University of Granada, Spain.

To assess sexual behaviors, questions were asked on the following: the age at which consenting vaginal sexual relations were first engaged in, the use of a condom during the first vaginal sex act, the number of persons with whom penetrative vaginal sex acts had been practiced in lifetime and in the previous 2 months, the frequency of vaginal sexual relations in the previous 2 months, and the frequency of penetrative vaginal sex acts using a condom (in the previous 2 months). Similar questions were asked to assess anal sex behavior.

#### Connor-Davidson Resilience Scale (CD-RISC) (Connor & Davidson, 2003)

We used the adaptation of this instrument to the Spanish population performed by Carvalho, Fernández-Calvo, Martín, Campos, and Castillo (2006). It is a self-administered questionnaire with 25 items, with a Likert response scale going from 0 “totally disagree” to 4 “totally agree,” which assesses resilience, handling stress, and recovery capacity. The maximum score is 100; the higher the score, the higher the resilience. The questionnaire is multi-dimensional and divided into five factors. Factor 1 assesses personal competence, high standards, and tenacity, made up of eight items (for example: “I try my hardest on every occasion”), with scores ranging from 0 to 32. Factor 2 assesses trust in intuition, tolerance to negative affect and tolerance to adversity, and is made up of seven items (for example: “I see the funny side of things”), with scores ranging from 0 to 28. Factor 3 assesses positive acceptance of change and is made up of five items (for example: “I can adapt to change”), with scores ranging from 0 to 20. Factor 4 assesses control through three items (for example: “I know where to go to get help”), with scores ranging from 0 to 12. Lastly, factor 5 assesses the influence of spirituality with two items (for example: “Sometimes I let fate or God guide me”), with scores ranging from 0 to 8. Internal consistency of 0.89 was observed in the original study and in the adaptation for the Spanish population (Carvalho et al., 2006; Connor & Davidson, 2003). In this study, the total internal consistency of the questionnaire was 0.90.

### Procedure

This research was approved by the Ethics Committee of the University of Granada (Spain). Data collection took place at the infectious-contagious diseases outpatient clinic of the San Cecilio Hospital of the University of Granada in Spain, using self-administered questionnaires, in the company of the researcher responsible for this study. All participants were undergoing treatment and medical follow-up at the hospital. Having explained the objectives of the study and having gained permission, a previously trained researcher went to the hospitals to carry out individual assessments. Informed consent was obtained from patients who participated in the study. All participants were informed that their participation was voluntary and that their replies would be anonymous and confidential. There was a 20% participation refusal rate. The refusal can be explained by the fact that at this hospital, there were other studies taking place at the same time, and this may have led to some participants feeling overburdened with questions and caused them to refuse. The assessment lasted approximately 20 min.

### Statistical analyses

Consistent use of a condom in vaginal sexual intercourse (in the previous 2 months) was calculated. To do this, first, the proportion of condom use in vaginal sexual intercourse in the previous 2 months was calculated, dividing the number of vaginal sexual relations using a condom by the total number of vaginal sexual relations. Then, the variable proportion of condom use in vaginal sexual relations over the previous 2 months was dichotomized, so that values equal to 1 indicated consistent use of condoms and values below 1 indicated inconsistent use of condoms. The same approach was used to calculate consistency of condom use in anal sexual intercourse (in the previous 2 months). To establish resilience levels (high, medium, and low), the 25th (low level) and 75th (high level) percentiles were considered. Scores between 25th and 75th percentiles were considered as medium level. We decided not to stratify the sample according to sexual orientation since it was a convenience and intentional sample, so the sample was not large enough for such analysis.

Student’s *T* test was used to compare hypotheses on two independent means and analysis of variance (ANOVA) when there were three or more levels of comparison. The Levene test was used to check whether population variances were equal. In cases where this condition was not met, we used Welch’s approximation to compare two independent means and the Brown-Forsythe test to compare three or more independent means. The Shapiro-Wilk test was used to check normality of distributions. In cases where this condition was not met and the number of sample subjects in a comparison group was small (*n* < 30), the Kruskal-Wallis non-parametric test was used for three or more independent medians. To analyze the effect of resilience on vaginal and anal sexual behaviors, hierarchical linear regression analysis and hierarchical logistic regression were carried out, controlling the effect of the socio-demographic variables. All the statistical analyses were carried out using the statistics package *SPSS* version 21.0.

## Results

### Socio-demographic variables and their relationship to resilience

The means and standard deviations in resilience factors according to socio-demographic variables were calculated (see Table 2). The total mean resilience of patients was 62.98. As regards total resilience levels, 27.68% had high resilience (*n* = 44), 49.05% had medium resilience (*n* = 78), and 23.27% had low resilience (*n* = 37).

**Table 2:** Means and standard deviations in resilience factors according to socio-demographic variables

Socio-demographic features	Factor 1	Factor 2	Factor 3	Factor 4	Factor 5	Total resilience
	Mean (SD)	Mean (SD)	Mean (SD)	Mean (SD)	Mean (SD)	Mean (SD)
Sex						
Male	20.00 (5.50)	15.71 (4.88)	14.31 (2.93)	7.81 (2.90)	3.97 (1.91)	62.80 (14.36)
Female	21.37 (5.99)	15.00 (5.06)	14.27 (2.47)	8.64 (2.47)	4.29 (2.03)	63.59 (14.63)
Sexual orientation						
Heterosexual	21.45 (6.03)	15.10 (4.87)	14.54 (2.68)	8.48 (2.77)	4.41 (1.94)	64.07 (14.21)
Homosexual	20.80 (5.29)	15.86 (4.95)	14.12 (3.06)	7.67 (2.83)	3.74 (1.90)	62.21 (14.21)
Age						
17 to 29 years old	20.30 (6.12)	14.69 (4.51)	14.04 (2.82)	8.08 (3.01)	4.13 (1.60)	61.26 (12.39)
30 to 42 years old	21.62 (5.22)	16.48 (4.74)	14.36 (3.10)	8.15 (2.70)	3.84 (1.98)	64.46 (14.37)
43 to 55 years old	20.81 (5.80)	14.94 (5.13)	14.32 (2.79)	7.85 (2.91)	4.21 (2.01)	62.15 (15.04)
Marital status						
Single	21.64 (5.18)	15.70 (4.90)	14.30 (3.17)	8.87 (2.76)	3.92 (1.76)	63.75 (13.85)
Married	20.22 (6.21)	14.93 (4.63)	14.67 (2.49)	7.48 (3.38)	4.58 (1.96)	61.90 (14.32)
Common-law spouse	20.13 (6.01)	15.86 (5.44)	14.00 (3.35)	8.04 (2.62)	3.72 (2.49)	61.77 (17.01)
Separated or divorced	20.64 (6.30)	14.50 (5.27)	13.57 (2.27)	7.57 (2.97)	3.71 (2.05)	60.00 (15.36)
Widow	21.69 (5.07)	16.61 (4.80)	14.69 (2.05)	8.69 (1.88)	4.46 (1.76)	66.15 (13.06)
Education level						
No schooling	21.00 (5.09)	13.00 (2.82)	14.33 (1.50)	7.33 (1.50)	4.33 (2.25)	60.00 (5.72)
Primary	20.70 (5.14)	15.12 (5.44)	14.14 (3.07)	8.10 (2.70)	4.10 (2.07)	62.16 (13.86)
Secondary	21.50 (5.94)	15.67 (4.87)	14.43 (2.95)	7.91 (3.21)	3.93 (1.83)	63.46 (15.54)
University	20.90 (5.83)	16.24 (4.53)	14.29 (2.88)	8.14 (2.55)	4.12 (1.96)	63.70 (14.36)
Income						
Less than 600 euros	21.10 (5.29)	15.30 (5.01)	13.60 (3.24)	7.90 (2.82)	3.83 (2.08)	61.73 (15.04)
600 to 900 euros	20.70 (4.93)	14.74 (3.95)	13.74 (2.80)	7.88 (3.04)	4.22 (1.80)	61.29 (12.01)
900 to 1200 euros	21.93 (4.60)	15.86 (4.48)	14.70 (2.53)	7.90 (2.91)	4.26 (1.91)	64.66 (11.66)
More than 1200 euros	20.84 (6.36)	15.81 (5.41)	14.63 (2.76)	8.15 (2.76)	9.98 (1.97)	63.44 (16.01)
Current employment situation						
Employed	20.74 (5.37)	15.33 (4.72)	14.13 (2.86)	8.10 (2.69)	3.94 (1.86)	62.25 (13.46)
Unemployed	21.24 (5.91)	16.42 (4.77)	14.64 (2.81)	7.73 (3.09)	3.99 (1.99)	64.00 (15.07)
On sick leave	22.00 (6.00)	14.70 (5.84)	14.29 (3.32)	8.20 (2.88)	4.62 (2.12)	63.83 (16.72)
Year of HIV/AIDS diagnosis						
1985–1990	23.04 (4.37)	15.66 (4.95)	13.57 (2.87)	8.85 (2.28)	4.76 (2.23)	65.90 (13.62)
1991–1995	22.10 (4.82)	16.24 (5.11)	14.62 (2.87)	8.51 (2.82)	4.37 (1.84)	65.86 (13.73)
1996–2000	20.50 (6.19)	14.27 (5.45)	13.77 (2.77)	8.05 (2.64)	3.00 (1.49)	59.61 (15.94)
Year of HIV/AIDS diagnosis						
2001–2005	20.55 (6.23)	15.41 (5.13)	14.96 (2.74)	8.00 (2.80)	4.27 (1.86)	63.20 (15.47)
2006–2010	20.33 (5.76)	15.61 (4.65)	14.24 (2.86)	7.48 (3.02)	3.85 (1.94)	61.53 (13.97)
Number of people to whom HIV status was disclosed						
None	23.07 (5.25)	14.92 (6.79)	15.50 (2.87)	8.21 (3.55)	4.07 (1.54)	65.78 (15.49)
1 to 5 persons	20.20 (5.65)	15.19 (4.53)	14.41 (3.09)	7.80 (2.92)	3.93 (2.09)	61.55 (13.92)
6 to 15 persons	20.74 (5.30)	15.58 (4.62)	13.64 (2.41)	7.89 (2.92)	3.82 (1.76)	61.69 (14.21)
16 to 30 persons	21.08 (5.36)	15.33 (4.16)	14.25 (2.34)	8.08 (1.72)	4.58 (1.97)	63.33 (12.15)
More than 30 persons	24.37 (5.58)	17.87 (6.09)	14.37 (3.44)	9.06 (2.64)	4.75 (1.91)	70.43 (16.53)
Total	21.07 (5.60)	15.54 (4.92)	14.30 (2.91)	8.01 (2.82)	4.05 (1.94)	62.98 (14.38)

*SD* standard deviation; *Factor 1* personal competence, high standards, and tenacity; *Factor 2* trust in intuition, tolerance to negative affect, and tolerance to adversity; *Factor 3* positive acceptance of change; *Factor 4* control; *Factor 5* spirituality

As regards sex, no significant differences in resilience were found between seropositive males and females. As regards sexual orientation, significant differences were observed only in the spirituality factor of resilience. Heterosexuals showed greater spirituality than homosexuals.

Looking at the year of HIV diagnosis, significant differences were found only in spirituality of resilience (factor 5) (*Kruskal-Wallis* = 9.59; *p* < 0.05). Patients who were diagnosed between 1985 and 1990 (between 23 and 28 years of diagnosis) showed greater spirituality than those diagnosed between 1996 and 2000 (13 and 17 years of diagnosis). As for the number of persons to whom patients had disclosed their seropositive status, significant differences were found only in the personal competence, high standards, and tenacity factor for resilience (factor 1) (Kruskal-Wallis = 9.38; *p* < 0.05). Patients who had disclosed their HIV-positive status to more than 30 persons had higher personal competence, higher standards, and higher tenacity than patients who had disclosed their HIV-positive status to between 1 and 5 persons.

### Resilience as predictor of vaginal and anal sexual behaviors

Table 3 shows the means and standard deviations for vaginal and anal sexual behaviors.

**Table 3:** Means and standard deviations in sexual behaviors

Variables	Participants (*N =* 159)
Age at first vaginal sex	*n* = 90
Mean	17.55
SD	3.19
Age at first anal sex	*n* = 51
Mean	23.70
SD	7.19
Number of vaginal sex partners (in lifetime)	*n* = 90
Mean	15.77
SD	20.85
Number of anal sex partners (in lifetime)	*n* = 50
Mean	19.54
SD	28.55
Number of vaginal sex partners (previous 2 months)	*n* = 46
Mean	1.36
SD	0.74
Number of anal sex partners (previous 2 months)	*n* = 27
Mean	3.22
SD	4.41

*SD* standard deviation

As regards consistency of condom use in vaginal sex, 89% used condoms consistently compared with 11% who used condoms inconsistently. For anal sex, 90% used condoms consistently compared with 10% who used condoms inconsistently.

Hierarchical linear regression analyses were performed to check whether resilience was a predictor of sexual behaviors (consistent use of condoms in vaginal and anal sex, age at first vaginal and anal sexual intercourse, number of vaginal and anal sex partners during lifetime and in the previous 2 months). The effect of socio-demographic variables was controlled.

Resilience was not shown to be a predictor of any sexual risk behaviors in vaginal sex: consistency of condom use in vaginal sexual relations in the previous 2 months (*F*_(5, 30)_ = 1.12; *p* = 0.37), age at first vaginal sexual relations (*F*_(5, 84)_ = 0.30; *p* = 0.87), and number of vaginal sex partners during lifetime (*F*_(5, 84)_ = 0.41; *p* = 0.84) and in the previous 2 months (*F*_(5, 40)_ = 0.50; *p* = 0.77).

Similarly, resilience was not a predictor of any sexual risk behaviors in anal sex: consistency of condom use in anal sex relations in the previous 2 months (*F*_(5, 52)_ = 1.37; *p* = 0.25), age at first anal sex relations (*F*_(5, 113)_ = 0.59; *p* = 0.70), and number of anal sex partners during lifetime (*F*_(5, 84)_ = 0.41; *p* = 0.84) and in the previous 2 months (*F*_(5, 67)_ = 0.87; *p* = 0.50).

Lastly, to analyze whether resilience was a predictive factor as regards the use of condoms in the first vaginal and anal sex acts, hierarchical logistic regression analyses were performed. Looking at condom use in the first vaginal sexual relations, the model was not found to be significant (*χ* ^2^_5_ = 3.96; *p =* 0.55; *R* ^2^ = 0.04). Neither was the model found to be significant for condom use in first anal sex act (*χ* ^2^_13_ = 4.91; *p =* 0.42; *R* ^*2*^ = 0.04).

## Discussion

Looking at the first objective of this study, almost half of the participants (49.05%) showed medium resilience and 27.68% showed high resilience. Corroborating this study, a previous study demonstrated that the participants had low level of resilience (Wilson et al., 2016). Some studies show that persons with HIV infection have a high capacity to adapt and recover (resilience) in spite of the adversities they face (De Santis et al., 2013; Kurtz et al., 2012; Munro & Edward, 2008). As regards sex, age group, education level, income, and current employment situation, no significant differences were found among patients. However, a recent study revealed that HIV-positive women with a low education level, low income, and no employment had high levels of resilience (Dale et al., 2014). Another previous study on asymptomatic seropositive men with low income and low education level showed that they had low levels of resilience (Lima et al., 2014). The differences revealed between these studies may be due to cultural differences and/or sample sizes.

As regards sexual orientation, heterosexual patients scored more highly in spirituality compared with homosexual patients. In recent studies, it can be observed that spirituality has helped PLHIV to cope with HIV (Ironson, Kremer, & Lucette, 2016; Pecoraro et al., 2016). Other studies have indeed shown that spirituality is a resource used to overcome the challenges presented by HIV (Hussen et al., 2014; Szaflarski, 2013). Spirituality in overcoming adverse events could therefore play an important role among persons with HIV infection/AIDS, particularly among heterosexuals.

In addition, patients who were diagnosed between 1985 and 1990 scored more highly for spirituality than patients diagnosed more recently. The group of persons whose HIV diagnosis dates from further back have lived through a period during which antiretroviral therapies had not been developed which, in tandem with their being older, might explain their greater reliance on spirituality as a means of support and coping. A recent systematic review showed that the diagnosis of HIV caused people to increase their spirituality as a way of coping with this disease (Araújo et al., 2015, Mishra, Togneri, Tripathi, & Trikamji, 2017). A relevant future research would be to investigate the association between spirituality and psychosocial variables.

Patients who had disclosed their seropositive status to more than 30 persons showed greater personal competence, higher standards, and greater tenacity than patients who had revealed their seropositive status to fewer persons (1–5 persons). The first group probably has a larger social support network, which would provide greater psychological protection and coping resources for the adversities of HIV. This group might also have better assimilated their seropositive status and feel less stigmatized and discriminated against, leading them to tell a larger number of people about their condition, thus facilitating their receiving more social support. Thus, it can be demonstrated that social support mediated the relationship between the disclosure of serological status and self-esteem and depression (Zea et al., 2005). In line with this idea, it has been concluded that social support contributes to higher resilience among persons with HIV (Carvalho et al., 2007). Moreover, lack of social support has been identified as a factor in the progression of HIV/AIDS (Schuster, Bornovalova, & Hunt, 2012).

Moving on to the second objective of this study, it was not found that resilience acts as a predictor of sexual behaviors among persons with HIV infection. This lack of significant association might be explained by the reduced number of patients who answered the questions on sexual behaviors. Another plausible explanation could be that patients did not perceive their sexual risk behaviors as adverse situations for infection and/or reinfection by STI/HIV. It is important to underline that for resilience to come about, persons must be exposed to difficult situations that threaten their physical and/or psychological health (De Santis et al., 2013), so it may be that this sample did not feel that sexual risk behaviors were a threat to their health and, therefore, there is no link between resilience and these behaviors. Finally, in a recent systematic review, we found studies that showed that resilience was a protective variable of mental health, satisfaction with PLHIV’s life (Lyons & Heywood, 2016), and risky sexual behavior for HIV (Araújo et al., 2015).

This study has significant implications. It contributes results on resilience among HIV-positive young people and adults, where a large proportion of articles published focus on resilience among healthy children (Araújo et al., 2015; De Santis et al., 2013). It does, however, suffer from some limitations, given that as patient recruitment was incidental, the results cannot be generalized to the population as a whole. Ultimately, it would be appropriate to undertake studies which look more closely at resilience from a mixed-methods perspective (qualitative and quantitative) as suggested by Ungar (2009), with the aim of understanding what such studies can contribute in subjective and objective terms to how persons with HIV infection build up resilience.

## Conclusions

In conclusion, this study increases our understanding of the association between resilience, socio-demographic variables, and sexual behavior of PLHIV. This study contributed to fill the gap of studies on resilience and HIV infection. Secondly, it was not possible to verify this construct as a predictor for risky sexual behaviors of PLHIV. Finally, it was found in this sample that living with HIV does not represent an adversity for these people.

However, this study has some limitations. Because it was a cross-sectional study, it is not possible to make causal inferences. In addition, given that as participant recruitment was incidental, the results cannot be generalized to the population as a whole. Thus, it would be interesting to carry out future studies with a larger, more representative sample of the HIV-positive Spanish population, as well as compare the different socio-cultural contexts.

It is also suggested that future studies may consider other variables (not contemplated in this study), such as health status, perceived health or perceived illness, and quality of life (Remor & Ulla, 2002) of PLHIV. Furthermore, it would be important to develop a specific tool to assess the resilience of PLHIV in order to measure levels of this construct within the context of HIV infection. The data from this study may support future psychosocial interventions to help PLHIV to cope with adversity, as well as prevent future risky sexual behaviors for HIV.
